# Evaluation of the quality of online patient information at the intersection of complementary and alternative medicine and hypertension

**DOI:** 10.1186/s40885-021-00193-z

**Published:** 2022-03-15

**Authors:** Jeremy Y. Ng, Jane Jomy, Alexandra Vacca

**Affiliations:** Department of Health Research Methods, Evidence, and Impact, Faculty of Health Sciences, McMaster University, Michael G. DeGroote Centre for Learning and Discovery, Room 2112, 1280 Main Street West, Hamilton, ON L8S 4K1 Canada

**Keywords:** Complementary therapies, Consumer health information / standards, Hypertension, Information services / standards, Internet, Patient education as topic / standards

## Abstract

**Background:**

Hypertension impacts 1.1 billion people globally; many patients seek complementary and alternative medicine (CAM), as a result of adverse side effects from antihypertensive medications or because they believe natural options are safer. The internet is increasingly playing a role in patient health information-seeking behavior, however, the variability of information quality across websites is unclear. Thus, the purpose of this study was to assess the quality of websites providing consumer health information at the intersection of hypertension and CAM.

**Methods:**

Four unique terms were searched on Google, across Australia, Canada, the US, and the UK. The first 20 webpages resulting from each search were screened for eligibility, and were included if they contained consumer health information relating to CAM therapies for the treatment/management of hypertension. To assess the quality of health information on eligible websites, we used the DISCERN instrument, a standardized quality index of consumer health information.

**Results:**

Of 90 unique webpages, 40 websites were deemed eligible and quality assessed. The 40 eligible websites were classified into seven categories: professional (*n =* 15), news (*n =* 11), non-profit (*n =* 5), health portal (*n =* 3), commercial (*n =* 2), government (*n =* 1), and other (*n =* 3). The mean summed DISCERN score was 52.34 (standard deviation [SD] *=* 10.69) out of 75 and the mean overall score was 3.49 (SD *=* 0.08) out of 5. A total of 10 websites had a total DISCERN score of 60.00 and above with an average rating of 4.33. Among these, Medicine Net (69.00) and WebMD (69.00) were determined to have the highest quality information. Websites generally scored well with respect to providing their aims, identifying treatment benefits and options, and discussing shared-decision making; websites generally lacked references and provided inadequate information surrounding treatment risks and impact on quality of life.

**Conclusions:**

While some websites provided high-quality consumer health information, many others provided information of suboptimal quality. A need exists to better educate patients about identifying misinformation online. Healthcare providers should also inquire about their patients’ health information-seeking behavior, and provide them with the guidance necessary to identify high-quality resources which they can use to inform shared-decision making.

## Background

Cardiovascular disease is a leading cause of death worldwide, with 18 million fatalities each year [1]. Hypertension, or persistent elevated blood pressure, is a primary risk factor for cardiovascular disease [2]. Hypertension is considered to be a “silent killer” as patients may not experience any immediate signs or symptoms even years after developing the condition [3]. Globally, the direct medical costs of hypertension are estimated to be $370 billion USD per year, with the health care savings from effective interventions projected to be $100 billion per year [4]. Conventional treatment for hypertension includes first line antihypertensive drugs such as thiazide diuretics, β-blockers, and angiotensin-converting enzyme inhibitors, which are used to promote the excretion of salt or relax the blood vessels [5, 6]. Though common, these pharmacological agents may lead to intolerable side effects including leg cramps, skin rash, loss of taste, palpitations, edema of the lower limbs, constipation, headaches, and dizziness [6, 7]. In an attempt to avoid such negative effects of pharmacological interventions for hypertension, CAM has become increasingly popular among patients [8–12].

CAM approaches are non-mainstream practices that are either used together or in place of conventional medicine, respectively [13]. Patients have cited that the reasons they seek CAM include dissatisfaction with conventional medicine and wanting symptom relief while avoiding the side effects associated with pharmaceutical medications [14–18]. In the United States, roughly 40% of adults use at least one type of CAM to treat a wide range of conditions [8]. For hypertension, about 70% individuals above 65 years of age report using at least one form of CAM therapy [19]. In fact, over 95 different types of CAM interventions have been identified for the treatment of hypertension, from natural products (such as herbs and garlic) to mind and body practices (such as relaxation and yoga) [20]. Despite the variety of modalities and the widespread use of CAM, there is comparatively a lack of research conducted on investigating if such treatments are indeed safer and have fewer side effects when compared to standard treatment [21].

The internet is an accessible source of health information, with 36.7% of people across the world accessing health related content [22]. Those who access these websites often claim that internet health information helps them to make decisions regarding their treatment options [11]. However, there is considerable concern regarding the quality, accuracy, and reputability of information available on the internet with regard to CAM therapies [18]. Concerns are largely attributable to the lack of regulation and standardization as authors are able to generate and share information online regardless of their qualifications and expertise [11]. The objective of this cross-sectional study is to examine the quality of CAM online consumer health information for the treatment/management of hypertension that a typical patient may access on the internet.

## Methods

### Search strategy and screening

A search was conducted to assess web-based information on CAM therapies for the treatment and/or management of hypertension that a typical user may find online. The most popular search engine holding nearly 90% of the search engine market share, Google, was used [12]. Perspectives from Canada (Google.ca), the United States (Google.com), the United Kingdom (Google.com.uk), and Australia (Google.com.au) were included to provide a more internationally representative search strategy. To mitigate any bias or influence on the results from previous search histories, browser history and cookies were erased using incognito mode on Google Chrome. The four following searches were developed by JYN and conducted by AV on May 3, 2020: “alternative medicine for hypertension”, “complementary and alternative medicine for hypertension”, complementary medicine for hypertension”, and “integrative medicine for hypertension”. For the purpose of this study, we defined CAM therapies as non-mainstream approaches that are used in place of conventional medicine, as per the National Centre for Complementary and Integrative Health (NCCIH): https://www.nccih.nih.gov/health/complementary-alternative-or-integrative-health-whats-in-a-name.

### Eligibility criteria

AV reviewed the first 20 webpages from each search for inclusion as user traffic drops by 95% after the first page of results [22]. Duplicates and ineligible webpages were then removed by AV. Eligible websites met the following inclusion criteria: 1) at least one webpage with CAM health information for hypertension, 2) information is publicly available without membership or subscription requirements, and 3) published in the English language. Websites were deemed not eligible if they met one or more of the following exclusions: inaccessible content due to broken links, peer-reviewed articles, books, videos, forums, online retailers, and eBook websites. If more than one eligible webpage was found from the same website, the overall website was evaluated for quality assessment using the DISCERN instrument.

### Data extraction and website quality assessment

JYN and AV data extracted the following items: URL, website type, types of CAM and non-CAM therapies, appearance in more than one search, and scores for qualitative features as outlined by the 16 items of the DISCERN instrument. The DISCERN instrument is a standardized quality index of consumer health information to allow health professionals, patients, and the general population to evaluate the quality of health information [23]. DISCERN can be used to judge the quality of a publication without the need of specialist knowledge and without reference to other publications or advisers. This questionnaire is divided into three sections to evaluate the qualities of treatment choices provided (questions 1 to 8), reliability of the source (questions 9 to 15), and overall information (question 16). Each question was scored using a Likert scale from 1 (lowest quality) to 5 (highest quality) [24]. Section 1 of the DISCERN instrument (questions 1 to 8) assesses the overall reliability of the information provided and determines whether the source can provide accurate information without being influenced by conflicts of interest. Section 2 (questions 9 to 15) assesses the quality of information surrounding the treatment choices and indicates whether the benefits, side-effects and mechanisms for the treatments presented are adequately discussed. Section 3 (question 16) highlights the quality rating of the information source as a whole for reliability and quality.

Once the eligible websites were identified, JYN and AV pilot tested the DISCERN instrument on three websites to standardize data extraction. Any discrepancies were resolved and following this, JYN and AV independently completed the data extraction and assessed the quality of health information on CAM for hypertension using DISCERN. Discrepancies were resolved without unduly modifying scores by all three authors in a collaborative fashion. JJ calculated the average of the two assessors’ scores for each question across all websites, providing an overall summed DISCERN score between 15 and 75, based on the scores for the first 15 questions. Additionally, JJ calculated the average score and SD for each DISCERN item along with an average score for all 16 items. Calculations were reviewed by all three authors.

## Results

### Search results

**Fig. 1:**
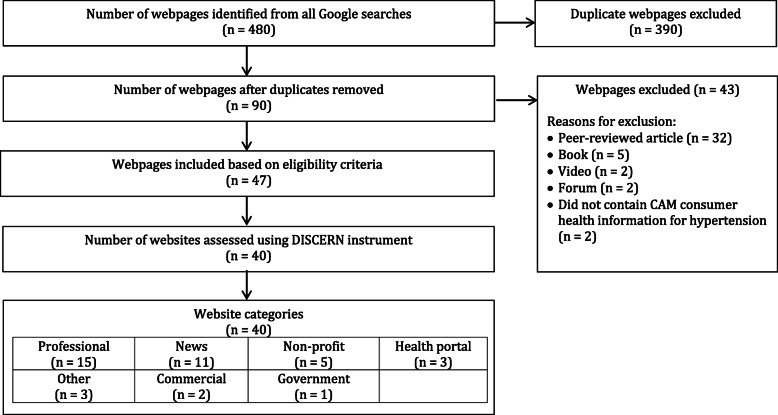
Web information search strategy and assessment flowchart. CAM, complementary and alternative medicine

A total of 480 webpages were identified through the Google searches, of which 390 were duplicates. Of the 90 unique webpages, 43 were excluded for the following reasons: peer-reviewed articles (*n* = 32), books (*n* = 5), videos (*n* = 2), forums (*n* = 2), and did not contain CAM consumer health information for hypertension (*n* = 2). Of the remaining 47 eligible webpages, seven were different webpages but from the same websites, leaving a final total of 40 websites which were assessed using the DISCERN instrument. The search strategy and assessment is summarized in Fig. 1.

### General characteristics

The 40 eligible websites were classified into seven categories: professional (*n* = 15), news (*n* = 11), non-profit (*n* = 5), health portal (*n* = 3), commercial (*n* = 2), government (*n* = 1), and other (*n* = 3). Professional websites were defined as those whose content was derived from health experts or managed by authorized institutions and organizations such as universities and hospitals. News websites included pages that provide relevant sources of consumer health information from newspapers, magazines, and television. Non-profit websites included those which were affiliated with charitable organizations and were not driven by financial incentives. Commercial websites comprised of those that were profit-oriented, with the intention of making sales. Government websites included pages regulated by a governing body. Websites that did not fit within any of these categories were classified as other.

Dietary and nutritional interventions, including the use of herbs and supplements, were the most commonly mentioned CAM therapies for hypertension, reported by all but one website (*n =* 39). Thirty-two websites discussed physical activity and weight loss or maintenance and 22 websites discussed mind and body therapies such as yoga, meditation, and breathing exercises. Additional CAM therapies discussed included physical therapy (*n* = 9), and stress reduction (*n* = 8). Non-CAM therapies for hypertension were mentioned by 25 websites, in which pharmacotherapy was the single most discussed intervention (*n* = 24), followed by self-monitoring of blood pressure (*n* = 6). The majority of websites appeared in more than one search, including searches using the same term for different countries (*n* = 33). Table 1 summarizes the general characteristics of the included websites.

**Table 1:** General characteristics of eligible websites

Website name	URL	Website category	Types of CAM discussed	Types of non-CAM therapies discussed	Appeared in more than one search?
Accredited Naturopathic Medical Schools	https://aanmc.org	Professional	Herbs, supplements, diet, acupuncture, meditation, biofeedback, physical activity	Pharmacotherapy	Yes
Allegheny Health Network	https://www.ahn.org	Professional	Acupuncture, massage, diet, wellness programs, weight loss, physical activity, sleep	Pharmacotherapy, self-monitoring	No
American Fitness Professionals & Associates	https://www.afpafitness.com	Other	Physical activity, diet, supplements, herbs	None	Yes
American Heart Association	https://professional.heart.org	Non-profit	Chiropractic/osteopathic interventions, massage, acupuncture, weight loss, physical activity, yoga, herbs, yoga, behavioural therapy	None	Yes
Avicenna	https://www.avicennaherbs.co.uk	Commercial	Weight loss, sleep, physical activity, yoga, tai chi, diet, relaxation, herbs, social counselling	None	No
Blood Pressure UK	http://www.bloodpressureuk.org	Non-profit	Herbs, yoga, meditation, supplement, weight loss, diet, physical activity	Pharmacotherapy, self-monitoring, medical counselling	Yes
British Health Foundation	https://www.bhf.org.uk	Non-profit	Physical activity, weight loss, diet	Pharmacotherapy	No
Cleveland Clinic	https://health.clevelandclinic.org	Professional	Diet, weight loss, physical activity, sleep, meditation, herbs, supplements	None	Yes
Clinical Advisor	https://www.clinicaladvisor.com	News	Diet, supplements	Pharmacotherapy, hand grip devices, deep breathing devices	Yes
Elcies	http://elcies.com	News	Diet, herbs, supplements, music, physical activity	None	Yes
Everyday Health	https://www.everydayhealth.com	News	Herbs, supplements	Pharmacotherapy	Yes
Florida Medical Clinic	https://www.floridamedicalclinic.com	Professional	Physical activity, diet, weight maintenance, relaxation, meditation,	Pharmacotherapy	Yes
FxMedicine	https://www.fxmedicine.com.au	Other	Supplements, nutrition	Pharmacotherapy	No
Greatlist	https://greatist.com	News	Weight maintenance, supplements, diet, meditation, deep breathing, relaxation	Pharmacotherapy	Yes
Harvard Medical School	https://www.health.harvard.edu	Professional	Weight loss, diet, physical activity, meditation, deep breathing	None	Yes
Health Line	https://www.healthline.com	Health portal	Physical activity, diet, weight loss, deep breathing, meditation, yoga,	Pharmacotherapy	Yes
Hypertension Institute	https://hypertensioninstitute.com	Professional	Spiritual wellness, spa, diet, weight management	Hormone therapy, biochemical tests, self-monitoring	No
iHealth Labs	https://ihealthlabs.com	Commercial	Physical activity, meditation, diet, supplement, stress management (work less)	Self-monitoring	Yes
Integrative Practitioner	https://www.integrativepractitioner.com	Other	Acupuncture	Pharmacotherapy	No
Johns Hopkins Medicine	https://www.hopkinsmedicine.org	Professional	Diet, weight loss, physical activity, stress management	None	Yes
Mayo Clinic	https://www.mayoclinic.org	Professional	Diet, physical activity, weight management	Self-monitoring, biochemical tests, pharmacotherapy, experimental therapies (catheter-based radiofrequency renal denervation, electrical stimulation of carotid sinus baroceptors)	Yes
Medical News Today	https://www.medicalnewstoday.com	News	Physical activity, diet, stress management (work less), music therapy, weight loss, meditation, deep breathing, supplements	None	Yes
Medicine Net	https://www.medicinenet.com	Health portal	Diet, weight loss, physical activity, deep breathing, muscle relaxation, mental imagery relaxation, music therapy, yoga, meditation, biofeedback, sleep, herbs, acupuncture, supplements	Pharmacotherapy, emergency injections	Yes
National Center for Complementary and Integrative Health	https://www.nccih.nih.gov	Government	Meditation, yoga, tai chi, qi gong, biofeedback, meditation, diet, supplements, herbs	Pharmacotherapy	Yes
Institute for Nature Medicine	https://naturemed.org	Non-profit	Diet, supplements, physical activity, stress management	Pharmacotherapy	Yes
Nursing Times	https://www.nursingtimes.net	News	Weight loss, diet, physical activity, relaxation exercises, herbs	Pharmacotherapy	No
Penn Medicine	https://www.pennmedicine.org	Professional	Physical activity, diet, stress management	None	Yes
Philadelphia Integrative Medicine	https://philly-im.com	Professional	Physical activity, diet, stress management, supplements	Pharmacotherapy	Yes
Physicians Weekly	https://www.physiciansweekly.com	News	Weight loss, diet, physical activity, biofeedback, meditation, acupuncture, slow breathing, yoga, relaxation techniques	None	Yes
Providence Health & Services	https://oregon.providence.org	Non-profit	Physical activity, weight loss, diet, supplements, yoga, meditation	Pharmacotherapy	Yes
St. Luke’s Hospital	https://www.stlukes-stl.com	Professional	Weight loss, diet, physical activity, supplements, herbs, homeopathy, acupuncture, massage/physical therapy, meditation, yoga	Pharmacotherapy	Yes
The Healthy	https://www.thehealthy.com	News	Physical activity, diet, herbs, meditation, animal therapy, sleep	Pharmacotherapy, self-monitoring,	Yes
Today’s Dietician	https://www.todaysdietitian.com	News	Supplements, physical activity, weight loss, diet	Pharmacotherapy	Yes
UC Health	https://www.uchealth.org	Professional	Acupuncture, traditional Chinese medicine, chiropractic therapy, herbs, supplements, massage, biofeedback, mindfulness exercises, diet, spiritual counselling	None	No
University of Rochester Medical Center	https://www.urmc.rochester.edu	Professional	Supplements, herbs, meditation, qi gong,	Pharmacotherapy, breathing & hand grip devices	Yes
University of Wisconsin	https://www.fammed.wisc.edu	Professional	Weight maintenance, physical activity, diet, meditation, slow breathing, biofeedback, supplements, herbs	None	Yes
Verywell Health	https://www.verywellhealth.com	News	Weight loss, diet, physical activity, herbs, supplements	Pharmacotherapy	Yes
Verywell Mind	https://www.verywellmind.com	News	Meditation, yoga, progressive muscle relaxation, slow breathing, music therapy, sex, diet, supplements	None	Yes
Vior	https://viorlife.com	Professional	Stress management, weight loss, physical activity, diet, supplements, herbs	Pharmacotherapy	Yes
WebMD	https://www.webmd.com	Health portal	Stress management, qi gong, slow breathing, meditation, tai chi, yoga, hypnosis, biofeedback, acupuncture, supplements, herbs	None	Yes

*CAM* complementary and alternative medicine

### DISCERN instrument ratings

**Fig. 2:**
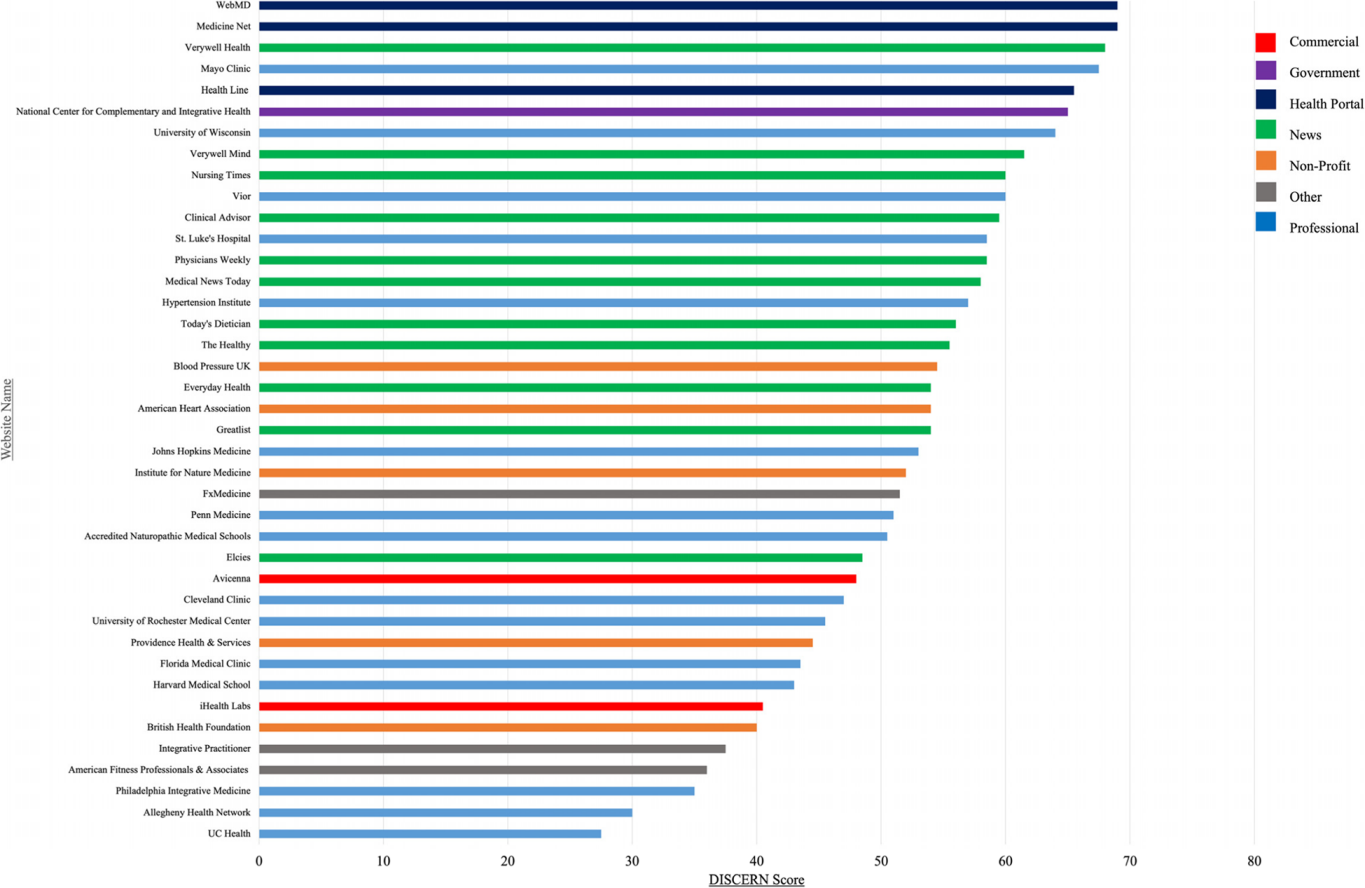
DISCERN scores by website category

The total DISCERN scores ranged from 27.50 to 69.00, out of 75.00. The mean score across all 40 websites was 52.34 (SD *=* 10.69). An average score of question 16 (overall assessment) was 3.49 (SD *=* 0.08) out of 5.00, indicating that the websites were overall moderate in quality. A total of 7 websites had a total DISCERN score of 63.00 and above with an average rating of 4.46. Among these, Medicine Net (69.00) and WebMD (69.00) were determined to have the highest quality information. In contrast, five websites had a DISCERN score of 37.50 and below with an average rating of 2.21. The two websites that scored the lowest were Allegheny Health Network (30.00) and UC Health (27.50). The summed DISCERN scores of all included websites are shown by category in Fig. 2.

Overall, the websites scored well on questions 1, 10, 14, and 15, and performed poorly on questions 4, 11, and 13 of the DISCERN assessment. A full breakdown of the DISCERN scores for each question and website is outlined in Table 2.

**Table 2:** DISCERN instrument ratings

Section		SECTION 1 Is the publication reliable?								SECTION 2 How good is the quality of information on treatment choices?							SECTION 3 Overall rating of the publication		
DISCERN question		1. Are the aims clear?	2. Does it achieve its aims?	3. Is it relevant?	4. Is it clear what sources of information were used to compile the publication (other than the author or producer)?	5. Is it clear when the information used or reported in the publication was produced?	6. Is it balanced and unbiased?	7. Does it provide details of additional sources of support and information?	8. Does it refer to areas of uncertainty?	9. Does it describe how each treatment works?	10.Does it describe the benefits of each treatment?	11. Does it describe the risks of each treatment?	12. Does it describe what would happen if no treatment is used?	13. Does it describe how the treatment choices affect overall quality of life?	14. Is it clear that there may be more than one possible treatment choice?	15. Does it provide support for shared decision-making?	16. Based on the answers to all of the above questions, rate the overall quality of the publication as a source of information about treatment choices	SD of Overall Score (Q16)	DISCERN Score (Sum of Q1-Q15)
Medicine Net	https://www.medicinenet.com	5.00	4.00	5.00	4.00	5.00	5.00	4.50	5.00	4.00	5.00	4.00	4.50	4.00	5.00	5.00	4.60	0.00	69.00
WebMD	https://www.webmd.com	5.00	5.00	5.00	4.00	5.00	5.00	4.50	5.00	3.00	4.00	4.00	5.00	4.50	5.00	5.00	4.60	0.09	69.00
Verywell Health	https://www.verywellhealth.com	5.00	5.00	4.00	4.00	4.50	5.00	4.50	5.00	3.00	5.00	4.00	4.00	5.00	5.00	5.00	4.53	0.00	68.00
Mayo Clinic	https://www.mayoclinic.org	5.00	4.50	5.00	4.00	5.00	5.00	3.50	5.00	4.00	5.00	3.00	5.00	3.50	5.00	5.00	4.50	0.05	67.50
Health Line	https://www.healthline.com	4.50	5.00	4.00	4.00	5.00	5.00	5.00	5.00	3.00	5.00	2.00	4.50	3.50	5.00	5.00	4.37	0.14	65.50
National Center for Complementary and Integrative Health	https://www.nccih.nih.gov	5.00	5.00	4.50	3.00	5.00	4.50	5.00	5.00	4.00	5.00	3.00	3.00	3.00	5.00	5.00	4.33	0.09	65.00
University of Wisconsin	https://www.fammed.wisc.edu	4.50	5.00	4.00	4.00	3.00	4.00	3.50	4.50	4.00	5.00	4.50	5.00	3.00	5.00	5.00	4.27	0.00	64.00
Verywell Mind	https://www.verywellmind.com	5.00	5.00	4.00	4.50	5.00	4.00	5.00	1.00	4.50	5.00	2.50	1.50	5.00	4.50	5.00	4.10	0.05	61.50
Vior	https://viorlife.com	4.50	5.00	4.00	4.50	5.00	4.00	4.00	3.00	2.50	5.00	1.50	5.00	2.00	5.00	5.00	4.00	0.09	60.00
Nursing Times	https://www.nursingtimes.net	5.00	5.00	3.00	3.50	4.50	3.00	4.00	5.00	5.00	5.00	5.00	1.00	3.00	5.00	3.00	4.00	0.00	60.00
Clinical Advisor	https://www.clinicaladvisor.com	5.00	4.50	3.50	4.50	5.00	4.50	4.00	4.50	3.00	5.00	3.00	2.00	1.00	5.00	5.00	3.97	0.14	59.50
Physicians Weekly	https://www.physiciansweekly.com	5.00	4.00	4.00	4.00	5.00	4.50	5.00	5.00	2.00	4.00	3.00	1.00	2.00	5.00	5.00	3.90	0.05	58.50
St. Luke’s Hospital	https://www.stlukes-stl.com	5.00	4.00	4.00	4.00	5.00	4.50	5.00	5.00	2.00	4.00	3.00	1.00	2.00	5.00	5.00	3.90	0.05	58.50
Medical News Today	https://www.medicalnewstoday.com	4.50	5.00	4.00	4.50	3.00	4.00	4.50	5.00	4.00	5.00	1.50	5.00	2.00	5.00	1.00	3.87	0.09	58.00
Hypertension Institute	https://hypertensioninstitute.com	5.00	4.50	4.50	1.00	2.00	2.00	3.50	5.00	3.00	4.00	3.50	5.00	4.00	5.00	5.00	3.80	0.09	57.00
Today’s Dietician	https://www.todaysdietitian.com	5.00	4.00	4.00	2.50	2.50	4.00	1.00	5.00	4.00	5.00	3.00	5.00	1.00	5.00	5.00	3.73	0.00	56.00
The Healthy	https://www.thehealthy.com	5.00	3.50	4.00	4.00	3.00	4.00	5.00	5.00	3.00	5.00	1.00	1.00	2.00	5.00	5.00	3.70	0.05	55.50
Blood Pressure UK	http://www.bloodpressureuk.org	4.50	5.00	4.00	1.00	2.00	3.00	3.00	4.50	2.50	4.00	4.00	3.50	3.50	5.00	5.00	3.63	0.05	54.50
Greatlist	https://greatist.com	3.50	5.00	3.50	5.00	5.00	4.00	3.00	4.50	3.00	4.50	1.50	1.50	2.50	5.00	2.50	3.60	0.00	54.00
American Heart Association	https://professional.heart.org	1.50	2.00	3.00	4.50	5.00	5.00	3.00	5.00	4.00	4.00	2.00	2.00	3.00	5.00	5.00	3.60	0.28	54.00
Everyday Health	https://www.everydayhealth.com	4.50	5.00	4.00	1.50	2.50	4.50	2.00	5.00	4.00	5.00	4.00	1.00	1.00	5.00	5.00	3.60	0.09	54.00
Johns Hopkins Medicine	https://www.hopkinsmedicine.org	5.00	4.50	4.50	1.00	1.00	3.00	3.00	4.00	3.00	4.00	2.50	4.50	3.00	5.00	5.00	3.53	0.00	53.00
Institute for Nature Medicine	https://naturemed.org	5.00	3.50	4.00	2.50	1.50	2.50	3.00	2.00	2.00	5.00	2.00	5.00	4.00	5.00	5.00	3.47	0.09	52.00
FxMedicine	https://www.fxmedicine.com.au	5.00	4.00	2.50	3.50	3.50	3.50	4.00	1.50	3.50	4.50	1.50	2.00	4.00	3.50	5.00	3.43	0.33	51.50
Penn Medicine	https://www.pennmedicine.org	5.00	3.50	3.00	1.50	2.50	4.00	4.00	1.00	2.00	4.00	3.00	4.00	4.00	4.50	5.00	3.40	0.19	51.00
Accredited Naturopathic Medical Schools	https://aanmc.org	4.00	3.00	3.00	2.50	2.50	2.00	1.50	5.00	4.00	5.00	2.50	3.00	3.00	5.00	4.50	3.37	0.05	50.50
Elcies	http://elcies.com	4.00	4.00	4.00	3.00	1.50	1.50	3.00	2.00	4.00	5.00	1.50	5.00	4.00	3.00	3.00	3.23	0.14	48.50
Avicenna	https://www.avicennaherbs.co.uk	5.00	4.00	4.00	1.00	1.00	4.50	1.00	3.50	3.00	4.00	1.50	3.00	2.50	5.00	5.00	3.20	0.00	48.00
Cleveland Clinic	https://health.clevelandclinic.org	5.00	3.50	4.50	3.00	2.50	2.50	3.00	4.50	3.00	5.00	1.50	2.00	1.00	5.00	1.00	3.13	0.28	47.00
University of Rochester Medical Center	https://www.urmc.rochester.edu	3.00	3.00	3.00	1.00	2.50	2.50	1.50	5.00	3.00	4.00	4.00	1.00	2.00	5.00	5.00	3.03	0.05	45.50
Providence Health & Services	https://oregon.providence.org	3.00	3.00	4.00	1.00	1.00	2.00	1.00	5.00	2.50	4.50	2.00	3.00	2.50	5.00	5.00	2.97	0.05	44.50
Florida Medical Clinic	https://www.floridamedicalclinic.com	2.50	3.00	4.00	1.00	2.50	3.00	1.50	1.50	2.00	4.00	1.50	4.50	2.50	5.00	5.00	2.90	0.05	43.50
Harvard Medical School	https://www.health.harvard.edu	5.00	3.00	3.50	1.00	2.50	4.00	1.50	1.50	2.50	4.00	1.00	1.50	2.00	5.00	5.00	2.87	0.00	43.00
iHealth Labs	https://ihealthlabs.com	4.00	3.50	3.00	1.00	2.50	2.00	1.50	1.00	3.00	4.00	2.00	5.00	2.00	5.00	1.00	2.70	0.05	40.50
British Health Foundation	https://www.bhf.org.uk	1.50	1.00	3.00	1.00	1.00	2.50	3.50	2.00	3.00	3.00	1.50	5.00	2.00	5.00	5.00	2.67	0.09	40.00
Integrative Practitioner	https://www.integrativepractitioner.com	5.00	3.00	2.00	1.00	2.50	3.00	5.00	4.00	1.00	3.50	1.50	1.00	1.00	3.00	1.00	2.50	0.14	37.50
American Fitness Professionals & Associates	https://www.afpafitness.com	4.50	4.50	3.00	1.50	2.50	2.00	1.50	1.00	3.00	4.00	1.00	1.00	1.50	3.00	2.00	2.40	0.09	36.00
Philadelphia Integrative Medicine	https://philly-im.com	4.50	2.00	2.50	1.00	2.50	2.00	3.00	1.00	2.50	4.50	1.00	2.00	1.00	4.50	1.00	2.33	0.09	35.00
Allegheny Health Network	https://www.ahn.org	4.00	3.00	3.00	1.00	1.00	1.50	1.00	1.00	1.00	1.00	1.00	1.00	1.00	4.50	5.00	2.00	0.00	30.00
UC Health	https://www.uchealth.org	3.00	2.00	2.00	1.00	1.50	1.50	1.50	1.00	2.00	2.00	1.00	1.00	1.50	1.50	5.00	1.83	0.14	27.50
**Total means**		4.38	3.90	3.69	2.64	3.13	3.45	3.19	3.61	3.04	4.34	2.41	3.03	2.63	4.68	4.25	3.49	0.08	52.34
**Total SD**		0.95	1.04	0.76	1.46	1.47	1.16	1.38	1.67	0.90	0.86	1.14	1.66	1.18	0.77	1.44	0.71	0.08	10.69

### Aims of websites

Question 1 of the DISCERN instrument assessed how clearly the website indicated its aims. The websites scored high for clarity with the mean score of 4.38 (SD *=* 0.95) out of 5.00, with 33 of the 40 websites (82.5%) scored 4.00 or above. Most websites addressed this information clearly on either their homepage or “About Us” page by outlining their mission statement, goals, target audience, and type of information provided.

### Sources and referencing

Question 4 of the DISCERN instrument evaluated whether websites provided a clear list of sources used to compile the information shared. A high score on this criterion signified both the presence of in-text citations and a reference list. The total mean score for this question was 2.64 (SD *=* 1.46), where 23 of the 40 websites (57.5%) scored 3.00 or below, indicating that more than half of the websites performed poorly in this area. Almost all websites had either embedded citations with no reference list, a reference list with no embedded citations, or no references at all.

### Benefits and risks of CAM treatments

Question 10 of the DISCERN instrument assessed to what extent the benefits of CAM treatments were discussed. In general, most websites scored highly on this item, with 36 of the 40 websites (90%) scoring 4.00 and above and 18 websites (45%) scoring perfectly. The mean total DISCERN score for this question was 4.34 (SD *=* 0.85). A good breadth of information was generally provided on the benefits of various CAM treatment options in the online resources we assessed.

In contrast, question 11 of the DISCERN instrument focused on the potential risks of CAM treatments. The mean total score for this question was 2.41 (SD *=* 1.14). Only one website achieved a score of 5.00, while 31 of the 40 websites (77.5%) scored 3.00 or below. Almost all websites did not adequately address the adverse side effects of various CAM treatments, while some did not mention this topic at all. We found that the websites had a tendency to preferentially report the health benefits of CAM treatments.

### CAM treatment impact on quality of life

Question 13 of the DISCERN instrument examined whether or not the websites discussed the impacts of CAM treatments on the patient's quality of life. The websites scored poorly on this item with only 12 of the 40 websites (30%) scoring higher than a 3.00 and only 2 websites receiving a perfect score. The total mean DISCERN score was 2.63 (SD *=* 1.18). We found that the websites commonly focused on the direct physical impacts of the suggested CAM therapies, but often failed to address the mental and social consequences which could influence patients’ quality of life.

### Diversity of CAM treatment options

Question 14 of the DISCERN instrument assessed to what extent various treatment options were discussed. The mean score of 4.68 (SD *=* 0.77) was found, where 31 of the 40 websites (77.5%) received a perfect score, with only one website receiving a score below 3.00. The assessed websites were generally adequate in providing a diversity of treatment options for hypertension. Commonly discussed CAM treatment options included dietary and herbal supplements, exercising, mindfulness practices, physical therapy, and stress management. Some non-CAM treatment methods discussed included pharmacotherapy, self-monitoring, experimental therapy, and assistive devices.

### Shared decision-making

Question 15 of the DISCERN instrument assessed if shared decision-making was encouraged by the websites. The total mean score for this item was 4.25 (SD *=* 1.44), and a perfect score was achieved by 30 of the 40 websites (75%). Only nine websites (22.5%) scored below 3.00. Most websites recommended that patients should consult with healthcare providers before taking dietary and herbal supplements, and to seek medical help should they feel unwell. Additionally, disclaimers were commonly found, advising users that the information should not be substituted for professional guidance.

### Recommended websites for patients and consumers

A list of the recommended websites for patients seeking information on CAM therapy for hypertension is provided in Table 3; this list was informed by the fact that information sources with a DISCERN score of 63 to 75 points are reported as “excellent” in the published medical literature [25]. These websites (*n =* 7) received an overall DISCERN score above 63.00 and an overall rating above 4.00. They consistently scored highly on questions 2, 8, 10, 14, and 15, in which at least five of the seven websites received a perfect score of 5. These websites scored highly due to the fact that they adequately addressing aims, treatment benefits, various treatment options, and the importance of shared-decision making. They also provided relevant information, aimed to reduce bias, and provided appropriate supporting references. Areas that these websites performed relatively weaker in were addressing the mechanisms and risks of treatments and providing additional sources of supporting information, as assessed by questions 7, 9, and 11.

**Table 3:** Recommended websites for patients and consumers

Website name	URL	DISCERN score (sum Q1–15)	DISCERN rating (Q16)	Website category	Target audience	Frequency of updates
Medicine Net	https://www.medicinenet.com/high_blood_pressure_treatment/article.htm	69.00	4.60	Health portal	Healthcare providers, researchers, patients/public	The precise frequency of updates is not available.
WebMD	https://www.webmd.com/hypertension-high-blood-pressure/guide/hypertension-complementary-alternative-treatments	69.00	4.60	Health portal	Healthcare providers, researchers, patients/public	Website states that its content is timely and credible but the precise frequency of updates is not available.
Verywell Health	https://www.verywellhealth.com/hypertension-treatment-1763942	68.00	4.53	News	Healthcare providers, patients/public	Website states that content is up-to-date but the precise frequency of updates is not available.
Mayo Clinic	https://www.mayoclinic.org/diseases-conditions/high-blood-pressure/diagnosis-treatment/drc-20373417	67.50	4.50	Professional	Healthcare providers, researchers, aspiring medical professionals, patients/public	Website states that content is updated regularly according to a schedule to reflect current/revised findings but the precise frequency of updates is not available.
Health Line	https://www.healthline.com/health/high-blood-pressure-home-remedies	65.50	4.37	Health portal	Healthcare providers, patients/public	Website states that there is a set maintenance schedule but articles are also updated when new information becomes available. A precise frequency of updates is not available.
National Center for Complementary and Integrative Health	https://www.nccih.nih.gov/health/hypertension-high-blood-pressure	65.00	4.33	Government	Healthcare providers, researchers, aspiring medical professionals & researchers, patients/public	Website states that some parts are updated daily while others may not be updated for weeks to months.
University of Wisconsin	https://www.fammed.wisc.edu/integrative/resources/modules/hypertension/	64.00	4.27	Professional	Healthcare providers, researchers, aspiring medical professionals, patients/public	The precise frequency of updates is not available.

## Discussion

Due to the increasing popularity of CAM therapies for hypertension and the ease of access of web-based CAM information, a need exists to assess the quality of online information available to potential patients [8, 11]. Content from the internet may lack credibility, yet they frequently guide patients’ decisions about treatment and care [8]. This is particularly concerning as many patients elect to use CAM therapies without consulting their physicians and other healthcare providers [26].

In the present study, we assessed a total of 40 websites that provided CAM consumer health information for the treatment/management of hypertension, in which the greatest number of websites were categorized as professional webpages (*n =* 15). Dietary and nutritional interventions were the most commonly discussed types of CAM, followed by physical activity and weight management. Overall, the included websites were of moderate quality. Among these, 7 websites were identified as high quality resources which may be of value to healthcare providers for recommendation to patients. While five websites scored below 50% (of 75.00), the majority of websites received a passing score but were suboptimal in quality. Websites generally scored well in the following items: addressing the aims, treatment benefits, importance of shared-decision making, and variety of treatment options. In contrast, items that scored poorly on the DISCERN assessment were addressing treatment risks, explaining impacts on quality of life, and providing adequate and credible references.

### Comparative literature

Though to our knowledge, this is the first study to examine the quality of online CAM consumer health information for hypertension, some previously published studies have investigated the quality of online information pertaining to hypertension or cardiovascular disease in general. Tahir et al. [27] examined the quality of general online health information for high blood pressure. In this study, the mean DISCERN score was 48.10 across 25 websites, deeming them as being fair in quality. Similar to the findings of the present study, both health professionals and lay reviewers of Tahir et al.’s study reported that references and supplementary sources were not provided by the majority of websites [27]. Oloidi et al. [28] assessed online health information related to angiotensin receptor blockers, a common pharmacological treatment for hypertension. The authors reported that their subset of assessed websites had an average overall DISCERN rating of 2.99 of 5 (SD *=* 1.05) with the majority of websites rated as being moderate in quality (66%). Similar to the current study, Oloidi et al. [28] found that the websites performed well in describing treatment benefits and supporting shared-decision making, but performed poorly in providing references and impacts on quality of life. In contrast to the present study, however, Oloidi et al. [28] found that the item pertaining to description of treatment risks scored highly. Lastly, Bastos et al. [29] examined the quality of online health information for acute myocardial infarction and stroke. The authors reported that more than half of the websites received a score of 1.00 for their overall DISCERN rating, deeming the websites as being low in trustworthiness. Overall, our results were comparable to these previous studies in that the websites were assessed to be moderate or suboptimal in quality. DISCERN scores across the current study were generally higher than these aforementioned studies, suggesting that sources of online information specific to CAM and hypertension may be of slightly higher quality when compared to that of cardiovascular disease or hypertension alone. 

We can also compare to previous studies which examined the quality of online information with respect to CAM. Contrary to our findings, one study looking at web-based information on herbal medication for cancer treatment found that the websites were overall poor in quality. The average DISCERN ratings of 2.35 out of 5.00 (SD = 0.57) and 2.02 out of 5.00 (SD = 0.51) were found for website quality and safety, respectively. Another distinction included the fact that the prior study found their subset of websites to score very low score for the item pertaining to the variety of treatment choices (1.74; SD = 0.87), which we found to score very high [11]. Nonetheless, the study identified that the websites scored poorly with respect to providing treatment risks, impacts on quality of life, and adequate referencing, while receiving a high score for the discussion of treatment benefits [11]. These trends are congruent with our findings. Similarly, another study which conducted a systematic search of web-based CAM information also found that websites comprehensively reported treatment benefits [18]. Lastly, a number of studies have investigated the quality of web information about CAM at the intersection of back pain [30], arthritis [31], neck pain [32], and type 2 diabetes [33]. In general, the results from each of these studies showed that the websites scored highly with respect to addressing the aims, treatment benefits, and treatment options, while lacking in the discussion of treatment risks and describing what would happen if no treatment was used, and to some extent the impacts on quality of life. These findings are largely comparable to that of the present study. Overall, the findings from the published literature is mostly in agreement with our findings although some distinctions exist. These differences may be attributable to variations in how the questions of the DISCERN instrument were interpreted by different researchers, as well as variations in the health topics that were studied.

### Implications for practice and research

The present study found that the majority of the included websites were suboptimal in quality, highlighting the importance of improving the health literacy of patients. Further, research has shown that low health literacy may negatively impact one’s ability to evaluate online health information [34]. A previous study indicated that individuals with low health literacy tend to assess online health information using non-established evaluation criteria which are more subjective [34]. Thus, patients should be provided with user-friendly eHealth assessment tools based on established evidence-based criteria [35]. Healthcare providers should also direct patients to high quality sources of web-based health information in order to aid their patients in making sound decisions about their health [28, 35].

Additionally, it is critical to inform healthcare providers of the important role of patient-provider communication in mediating online health information usage [35]. Research has found that patients who viewed their care as being less patient-centered were more likely to seek and trust health resources they found online [36]. Thus, it is imperative that healthcare providers take an active role in building rapport with their patients in order to learn about their needs and guide their navigation of online information [36]. Interestingly, a study has found that the majority of healthcare providers lacked confidence in their ability to recommend the safety and accuracy of safe and accurate online information and had a limited knowledge of existing web resources that are of good quality. Therefore, healthcare provider education surrounding online resources through continuous professional development is important and necessary [37].

Since the greatest number of websites included in the present study were categorized as professional and as such, developed by health practitioners or academic institutions, a need exists to standardize and improve the quality of online information being presented and made available to patients [30]. For example, it would be prudent for health professionals should create online content based on high-quality clinical practice guidelines, which provide information that is evidence-based and updated regularly [5, 30]. For instance, Hypertension Canada’s 2018 Guidelines is an example of a good resource which outlines many non-conventional treatment methods while providing specific numerical targets for patients to abide by [5]. With these measures in place, there is considerable potential for improvement in patients’ experiences with online health information.

### Strengths and limitations

To our knowledge, it was the first study examining the quality of online CAM information for the treatment of hypertension, thus providing insight into the quality of information previously unassessed. In addition, the DISCERN instrument is a standardized instrument shown to be both reliable and valid for the purpose of assessing patient health information [23]. Our study was also strengthened by the fact that two authors assessed the subset of websites using the DISCERN instrument independently and in duplicate, and all three authors reviewed both sets of scores to mitigate bias. Moreover, we examined websites following conducting searches from the perspectives of four different countries, therefore improving the generalizability of our findings.

 Our study was restricted to assessing English websites, however, it cannot be denied that many users may access online resources in other languages as well. We also acknowledge that the pre-established search queries used in this study may not reflect those used by patients in the real world because layered searches and individually-selected terms may be utilized, thus yielding different search results. Lastly, our study did not examine quality differences across different categories of websites, nor is the DISCERN instrument capable of assessing the accessibility, utility, readability, and accuracy of the online resources. These factors are also important to consider as they are determinants of how readily a potential patient can obtain, understand and utilize the information they access.

## Conclusions

This cross-sectional study evaluated the quality of websites providing consumer health information at the intersection of CAM and hypertension using search queries to mimic the search strategy of a patient with hypertension. Websites were evaluated using the DISCERN instrument by two independent assessors to mitigate bias. Our results indicate that the majority of websites were moderate but suboptimal in quality and performed scored poorly with respect to the following items: providing treatment risks, impacts on quality of life, and credible references. Thus, it is critical that efforts are made to increase the health literacy of patients so that they can better evaluate the information they access on the internet. Further to this, healthcare providers should foster improved communication with patients with respect to, and become aware of, high-quality online resources that are available. With respect to the creation of future online patient resources, website developers should consider the use of current and high-quality evidence-based resources, such as clinical practice guidelines.

## Data Availability

All relevant data are included in this manuscript.

## Abbreviations

CAM: Complementary and alternative medicine
SD: Standard deviation
